# Treatment of infected lungs by ex vivo perfusion with high dose antibiotics and autotransplantation: A pilot study in pigs

**DOI:** 10.1371/journal.pone.0193168

**Published:** 2018-03-05

**Authors:** Norman Zinne, Marcus Krueger, Doris Hoeltig, Burkhard Tuemmler, Erin C. Boyle, Christian Biancosino, Klaus Hoeffler, Peter Braubach, Taufiek K. Rajab, Anatol Ciubotaru, Judith Rohde, Karl-Heinz Waldmann, Axel Haverich

**Affiliations:** 1 Department of Cardiothoracic, Transplantation, and Vascular Surgery, Hannover Medical School, Hannover, Lower Saxony, Germany; 2 Clinic for Swine, Small Ruminants, Forensic Medicine, and Ambulatory Service, University of Veterinary Medicine Hannover, Hannover, Lower Saxony, Germany; 3 Clinic for Paediatric Pneumology, Allergology, and Neonatology, Hannover Medical School, Hannover, Lower Saxony, Germany; 4 Biomedical Research in Endstage and Obstructive Lung Disease Hannover (BREATH), Member of the German Center for Lung Research, Hannover, Lower Saxony, Germany; 5 Institute for Pathology, Hannover Medical School, Hannover, Lower Saxony, Germany; 6 Department of Surgery, Brigham and Women’s Hospital and Harvard Medical School, Boston, Massachusetts, United States of America; 7 Department of Infectious Diseases, Institute for Microbiology, University of Veterinary Medicine Hannover, Hannover, Lower Saxony, Germany; Laurentian, CANADA

## Abstract

The emergence of multi-drug resistant bacteria threatens to end the era of antibiotics. Drug resistant bacteria have evolved mechanisms to overcome antibiotics at therapeutic doses and further dose increases are not possible due to systemic toxicity. Here we present a pilot study of ex vivo lung perfusion (EVLP) with high dose antibiotic therapy followed by autotransplantation as a new therapy of last resort for otherwise incurable multidrug resistant lung infections. Severe *Pseudomonas aeruginosa* pneumonia was induced in the lower left lungs (LLL) of 18 Mini-Lewe pigs. Animals in the control group (n = 6) did not receive colistin. Animals in the conventional treatment group (n = 6) received intravenous application of 2 mg/kg body weight colistin daily. Animals in the EVLP group (n = 6) had their LLL explanted and perfused ex vivo with a perfusion solution containing 200 μg/ml colistin. After two hours of ex vivo treatment, autotransplantation of the LLL was performed. All animals were followed for 4 days following the initiation of treatment. In the control and conventional treatment groups, the infection-related mortality rate after five days was 66.7%. In the EVLP group, there was one infection-related mortality and one procedure-related mortality, for an overall mortality rate of 33.3%. Moreover, the clinical symptoms of infection were less severe in the EVLP group than the other groups. Ex vivo lung perfusion with very high dose antibiotics presents a new therapeutic option of last resort for otherwise incurable multidrug resistant pneumonia without toxic side effects on other organs.

## Introduction

Ventilator-associated pneumonia (VAP) is currently the most commonly-acquired infection in intensive care units and represents a major cause of morbidity and mortality. The mortality rate for VAP ranges from 24% to 50% and can exceed 70% if the pneumonia is caused by high-risk pathogens[1–3]. *Pseudomonas aeruginosa* is one of the most frequent pathogens responsible for VAP[4] and is associated with a high mortality rate[5]. The development of resistance of *P*. *aeruginosa* to antibiotics is increasing globally and occurs as a result of various mechanisms, e.g. blocking drug penetration, efflux pumps, or modification of drug targets[6–8]. Since multidrug resistance, especially in Gram-negative bacteria, is one of the major concerns in current treatment strategies for pneumonia, new therapeutic alternatives are urgently needed[2,5,9].

Polymyxins are the most frequently used antibiotics capable of providing good coverage for *P*. *aeruginosa* strains resistant to beta-lactams and fluorquinolones[4]. Colistin is recognized as the only antibacterial drug that is potentially effective against many multidrug resistant strains of *P*. *aeruginosa*[2,8]. Its bactericidal effect is dose-dependent with >40 μg/ml being 100% lethal to *P*. *aeruginosa* in vitro[10]. Unfortunately, intravenous dose escalation is associated with non-tolerable systemic side effects, such as nephrotoxicity and neurotoxicity[11–13]. Aerosolized colistin is routinely used in patients with cystic fibrosis and has become a treatment option in VAP resulting from *P*. *aeruginosa* infection[14].

Initial clinical experience with ex vivo lung perfusion (EVLP) systems in lung transplantation has demonstrated the capacity to support organ preservation under near physiological conditions for up to 12 hours[15]. For ELVP, we use the Organ Care System™—a portable system used during lung transplantation that significantly reduces the cold ischemic insult to lung tissue and provides sustained perfusion and ventilation throughout transport from donor hospital to the recipient center. The Organ Care System™ provides normothermic perfusion and lung parenchyma-protective ventilation and potentially allows the assessment and optimization of organ function prior to transplantation[16–19]. EVLP has recently been explored as a means of ex vivo therapy for end-stage lung disease. A key step needed for implementation of ex vivo therapy is the ability to perform autotransplantation. We have recently described a surgical method for porcine pulmonary autotransplantation[20].

Pigs have previously been used to model *P*. *aeruginosa* lung infection[21,22]. We hypothesized that an ex vivo ventilation and perfusion system applied to porcine lungs would enable the targeted delivery of a very high dose of colistin, whilst avoiding the related systemic side effects. We therefore used this EVLP system to investigate the effectiveness of ex vivo very high dose colistin treatment of *P*. *aeruginosa* pneumonia in an autotransplantation animal model.

## Material and methods

### Animals

This study was conducted in accordance with the German Animal Welfare Act and the European Communities Council Directive 86/609/EEC for the Protection of Animals used for Experimental Purposes. All experiments were approved by the Local Institutional Animal Care and Research Advisory Committee and permitted by the local authorities (Lower Saxony State Office for Consumer Protection and Food, Animal Welfare Service, Permit Number: 33.9-42502-04-12/0919). Eighteen miniature pigs (Mini-Lewe), bred at the University of Veterinary Medicine Hannover, Germany, were used. The animals weighed 34±7 kg. Animals with anatomical variants that made successful autotransplantation impossible were excluded. All animals underwent virological and bacteriological screening for specific antibodies before being approved for the study.

### Bacteria

The well-characterized clinical isolate *P*. *aeruginosa* TBCF 10839 was originally isolated from a cystic fibrosis patient’s respiratory secretions [23]. *P*. *aeruginosa* TBCF 10839 showed sensitivity for in vivo patient treatment to colistin with a minimum inhibitory concentration (MIC) of 2 μg/ml and is resistant to amoxicillin (MIC 128 μg/ml).

### Pneumonia model

To reproducibly establish robust pneumonia in all animals, pigs were intramuscularly (i.m.) administered 16 mg/day dexamethasone (4 mg/ml, Vetoquinol, Ravensburg, Germany) for 3 days prior to infection to induce immune suppression[24]. Prior to infection, *P*. *aeruginosa* strain TBCF 10839 was cultured from a frozen stock on Columbia sheep blood agar at 37°C overnight. Bacteria were subcultured onto LB agar and grown overnight at 37°C. On the day of infection, plates were washed with 0.85% NaCl and a 10 ml suspension containing 3 x 10^9^ CFU in saline was administered bronchoscopically into the LLL of all animals[23].

### Therapy

Therapy began 24 hours after infection. Pigs were divided into three groups, with 6 animals each. The animals in Group A did not receive colistin treatment. The animals in Group B received daily intravenous (i.v.) application of 2 mg/kgBW colistin (CP-Pharma, Burgdorf, Germany). In Group C, lung explantation with subsequent ex vivo therapy and autologous re-transplantation was performed[20]. Pigs in Group C were anesthetized with an i.m. ketamine injection of 20 mg/kgBW (100 mg/ml, CP-Pharma, Burgdorf, Germany) and 2 mg/kgBW azaperone (Stresnil®, Elanco, Bad Homburg, Germany). Animals were intubated with a 7.5 mm endotracheal tube and relaxed with 6 mg of pancuronium bromide (Pancuronium-Actavis™, Actavis GmbH, Munich, Germany) given intravenously. Anesthesia was maintained with 2–3% isoflurane (Isoflo®, CP-Pharma, Burgdorf, Germany)[25]. For hemodynamic monitoring and blood sampling, an arterial catheter was inserted in the left carotid artery and a venous catheter was inserted in the left jugular vein. Pigs were monitored via capnometry, electrocardiography, and continuous arterial pressure measurements during the entire duration of the anesthesia.

**Fig 1:**
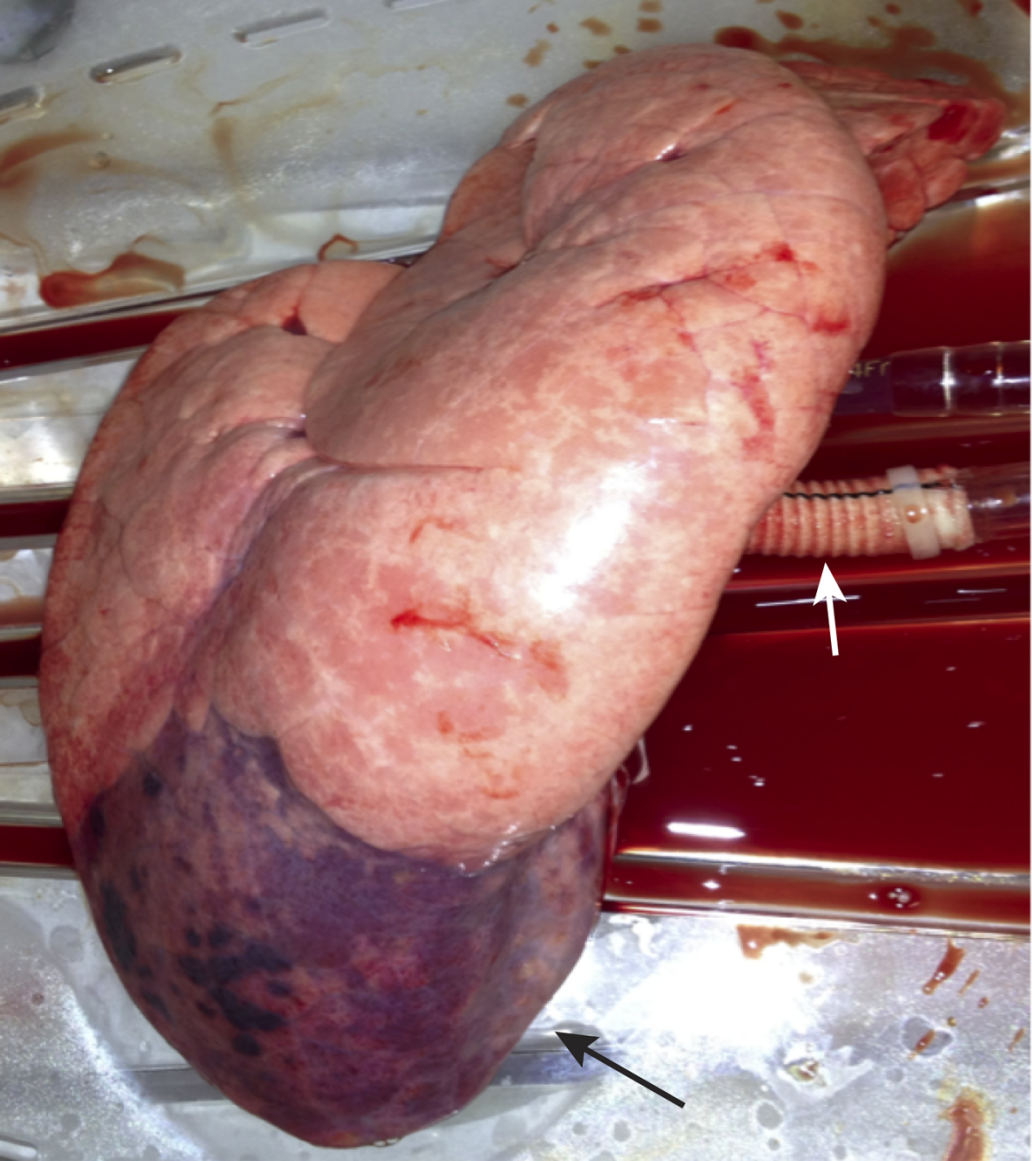
Implantation of an infected LLL into the EVLP system. Severe pneumonia of an explanted LLL (black arrow) 16 h after infection with *P*. *aeruginosa*. The left pulmonary artery and bronchus (white arrow) were elongated with a vascular prostheses and connected to the EVLP system.

A left-sided thoracotomy (5^th^ intercostal space) was performed under aseptic conditions, followed by the preparation of the left pulmonary artery, veins, and bronchus. After clamping the veins, artery, and bronchus, the infected LLL was explanted and flushed with 1 l of Perfadex® solution (XVIVO Perfusion, Goeteborg, Sweden) and buffered with 0.3 ml of Tris(hydroxymethyl)-aminomethane (THAM, Tris™ 36.34%, B. Braun Melsungen AG, Melsungen, Germany). In order to connect the lung to the EVLP system, elongation of the pulmonary artery and the bronchus was performed with two polyethylene terephthalate vascular prostheses (Gelweave™, Vascutek Terumo, Inchinnan, Scotland) (Fig 1).

The perfusion solution consisted of 2000 ml albumin-containing STEEN Solution™ (XVIVO Perfusion, Goteborg, Sweden), 1000 ml porcine blood, 500 mg methylprednisolone (Urbason®, Sanofi Aventis GmbH, Frankfurt am Main, Germany), one unit vial of multivitamins (Soluvit® N, Co. Baxter, Platting, Germany), 1 g glucose (Glucose 40%, B. Braun Melsungen AG, Melsungen, Germany), 1000 mg amoxicillin, and 800 mg colistin. The perfusion solution was applied at an initial temperature of 28°C. Ventilation of the lung was initiated at 32°C, 18 ±3 min after explantation. The lung was perfused for two hours at 37°C. The lung was then cooled to 14°C with ventilation stopping at 32°C. Afterwards, the lung was disconnected from the EVLP system, flushed with 1 l of Perfadex® solution, buffered again with 0.3 ml of THAM, and re-implanted into the same animal. Before the chest was closed, a thoracic drain was inserted.

All animals underwent the same daily medication protocol which included 2 mg/kgBW i.v. carprofen (Rimadyl®, Zoetis, Vienna, Austria), 0.4 mg/kgBW i.v. dexamethasone, twice daily applications of 0.02 mg/kgBW i.v. buprenorphine (Buprenovet Multidose®, Bayer Animal Health Care AG, Leverkusen, Germany), and 20 mg/kgBW i.v. amoxicillin (Amoxisel®, Bela-Pharm GmbH und Co., Vechta, Germany). All animals were examined by a veterinarian at least three times a day. The experiments were terminated 5 days post-infection (ie. 4 days post therapy), or earlier if clinically indicated. For humane endpoints the following criteria were used: a) mouth breathing, pulmonary edema (foamy nose and mouth discharge), and onset of low temperature (<38.5°C) after previous fever phase, b) fever (>40.7°C) and a refusal to feed for more than 3 days, c) a highly disturbed general condition, expressions of pain, and a refusal to feed for more than 3 days, d) evidence of circulatory failure (mouth breathing, cyanosis, cardiac arrhythmia). If animals reached the endpoint criteria, euthanasia was immediately performed. Animals were sacrificed using 80 mg/kgBW i.v pentobarbital (Euthadorm®, CP-Pharma, Burgdorf, Germany).

### Clinical scoring

To compare the severity of clinical symptoms, we used an optimized clinical score devised by Hoeltig based on respiratory rate, breathing type, breathing sound, cough, skin color, posture, behavior, food intake, and body temperature[26]. Animals were assessed once a day and given a score between 0 and 5. The more severe the clinical symptoms, the higher the score. After 5 days, each animal was given a cumulative score, with the maximum being 25.

### Pathology and microbiological assessment

Upon necropsy, lung lesion scores were determined according to the method described by Hannan et al. [27]. Briefly, the size and position of lesions were mapped on a diagram representing the seven lung lobes, with each lobe allotted a maximum possible score of 5. Then, by assessing the pneumonic area for each lobe as a fraction of 5 (resulting in a maximum score of 35 for the complete lung), the lung lesion score was calculated.

Tissue samples from each of the lung lobes were taken for histopathological and microbiological examination. For histopathological analyses, tissues were formalin-fixed and paraffin embedded using standard protocols. For evaluation, 4 μm thick tissue sections were stained with hematoxylin and eosin. Inflammatory changes in the lung parenchyma and the conducting airways were semi-quantitatively assessed. For microbiological examination, a semi-quantitative re-isolation score of *P*. *aeruginosa* was performed using a method similar to Maas et al. [28]. Each lung lobe was given a score between 0 and 3, with the maximum score being 21 for all 7 lobes. A reisolation score of 0 = no growth of *P*. *aeruginosa*; 1 = growth only in the inoculated area; 2 = growth in the inoculated area plus the first streaking; and 3 = growth in inoculated area and the first and second streaking.

### Statistics

Kruskal-Wallis one-way ANOVAs with Dunn’s post tests were performed using a 95% confidence interval. The results were expressed as mean values with standard deviations. Statistical analyses were performed using GraphPad Prism V5.

## Results

### Survival

**Fig 2:**
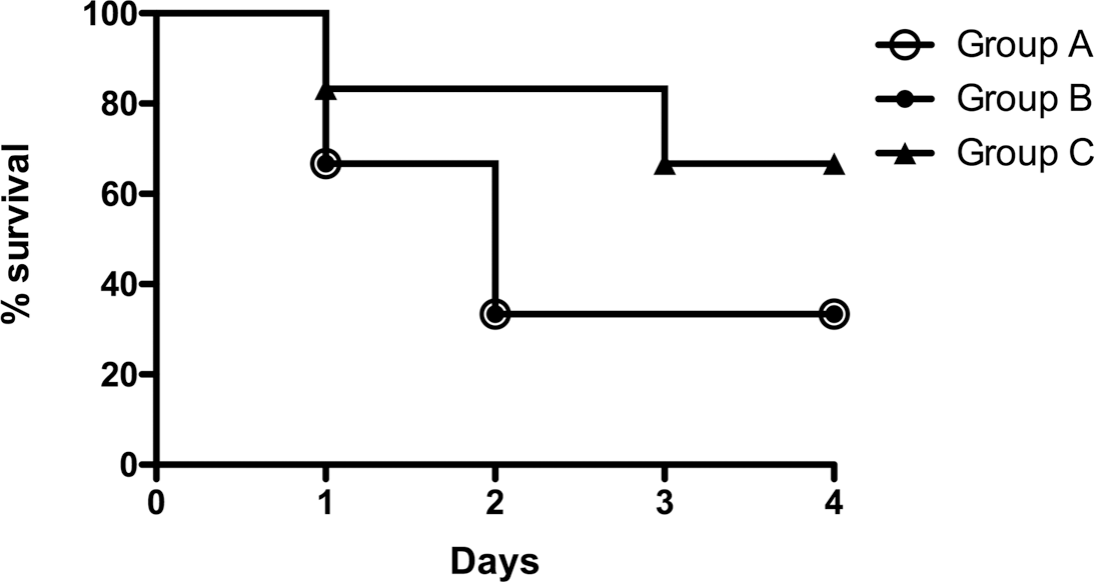
Survival of pigs with acute bacterial pneumonia given different therapies. *P*. *aeruginosa* pneumonia was induced in the LLL of 18 pigs. Twenty-four hours after infection, 6 pigs were not treated (Group A), 6 pigs were given i.v. colistin (Group B), and 6 pigs were treated for 2 hours by EVLP with high dose colistin followed by autotransplantation (Group C).

*P*. *aeruginosa* pneumonia was successfully induced in all animals. This study compared no treatment (Group A), conventional i.v. antibiotic treatment (Group B), and the novel approach of EVLP with high dose antibiotics followed by autotransplantation (Group C). In Group A, two animals had to be euthanized within the first 24 h after infection and two further animals within the period of 24 h to 72 h after infection due to severe clinical symptoms including fever, severe tachypnea, and hypoxia (Fig 2). The survival rate in Group B was comparable with Group A in that two animals had to be euthanized within 24 h, two further animals within the next 48 h. Therefore, only two animals in each group survived the 4 day post-infection period. Accordingly, the mortality in Group A and B was 66.7% (Table 1). In Group C, four animals survived the scheduled 4 day post-operative period. One animal had to be euthanized within the first 24h due to severe clinical symptoms (agitation, a paresis of the left foreleg, tachycardia). After an initially uneventful recovery period, a second animal in this group died after 48 h due to a volvulus, resulting in bowel rupture. Therefore, the infection-related mortality rate in Group C was 16.7%. Taking procedure-related mortality into consideration, the overall mortality rate was 33.4% (Table 1).

**Table 1:** Mortality rate of pigs with acute bacterial pneumonia given different therapies.

	Group A No therapy	Group B i.v. therapy^a^	Group C EVLP / AT^b^
Total	**6**	**6**	**6**
Death 1st day after infection	**2**	**2**	**1**
Death 2nd– 3rd day after infection	**2**	**2**	**1**
Euthanasia after 4th day (endpoint)	**2**	**2**	**4**
Infection-related mortality	**66.6%**	**66.6%**	**16.7%**
Procedure-related mortality	**-**	**-**	**16.7%**
Overall mortality	**66.6%**	**66.6%**	**33.4%**

^a^ Intravenous therapy of 2 mg/kg BW colistin.

^b^ EVLP with 200 μg/ml colistin for 2 hours followed by autotransplantation (AT).

### Clinical score

**Fig 3:**
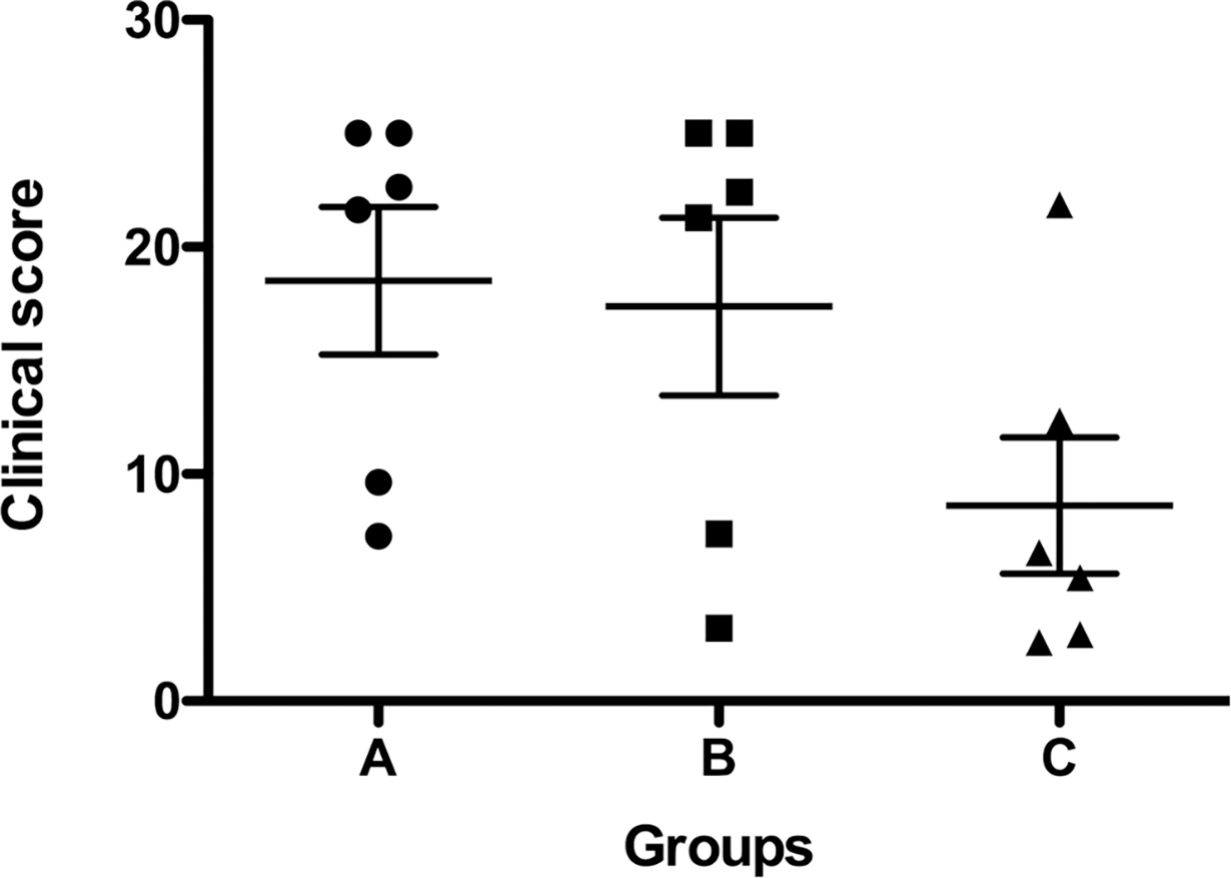
Clinical scores over the 5 day experimental period based on severity of clinical symptoms. *P*. *aeruginosa* pneumonia was induced in the LLL of 18 pigs. Twenty-four hours after infection, pigs were either not treated (Group A), given i.v. colistin treatment (Group B), or treated for 2 hours by EVLP with high dose colistin followed by autotransplantation (Group C).

Animals were assessed over the 5 day experimental period and given a daily clinical score based on respiratory rate, breathing type, breathing sound, cough, skin color, posture, behavior, food intake, and body temperature (Fig 3). In the case of death, animals were given the maximum score of 5 for the day they died and for all subsequent experimental days. Despite undergoing a major surgical procedure, animals in Group C had less severe clinical symptoms than animals in Group A and B (Fig 3).

### Macroscopic lung lesion score

**Fig 4:**
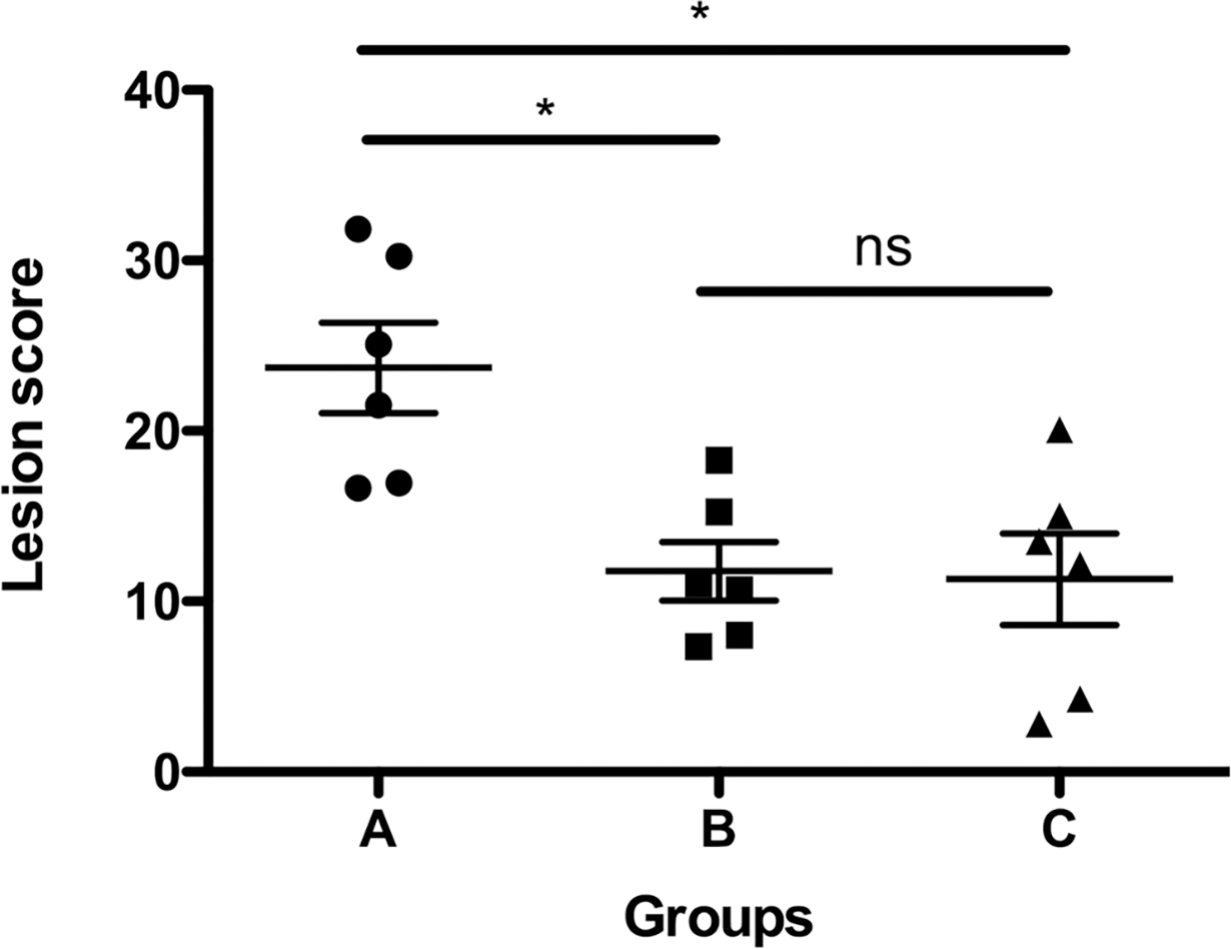
Macroscopic lung lesion score at necropsy. *P*. *aeruginosa* pneumonia was induced in the LLL of 18 pigs. Twenty-four hours after infection, pigs were either not treated (Group A), given i.v. colistintreatment (Group B), or treated for 2 hours by EVLP with high dose colistin followed by autotransplantation (Group C). *P < 0.*05*; ns (not significant) = *P > 0*.*05*.

To quantify the macroscopic pathological changes to the lung parenchyma, a lung lesion was performed upon necropsy. No pneumonic changes of the right lobe were found during necropsy. The pneumonia was always limited to the LLL. Both conventional i.v antibiotics (Group B) and ex vivo high dose antibiotics followed by autotransplantation (Group C) showed significantly less lesions when compared to no treatment (Group A) (Fig 4).

### Re-isolation score

**Fig 5:**
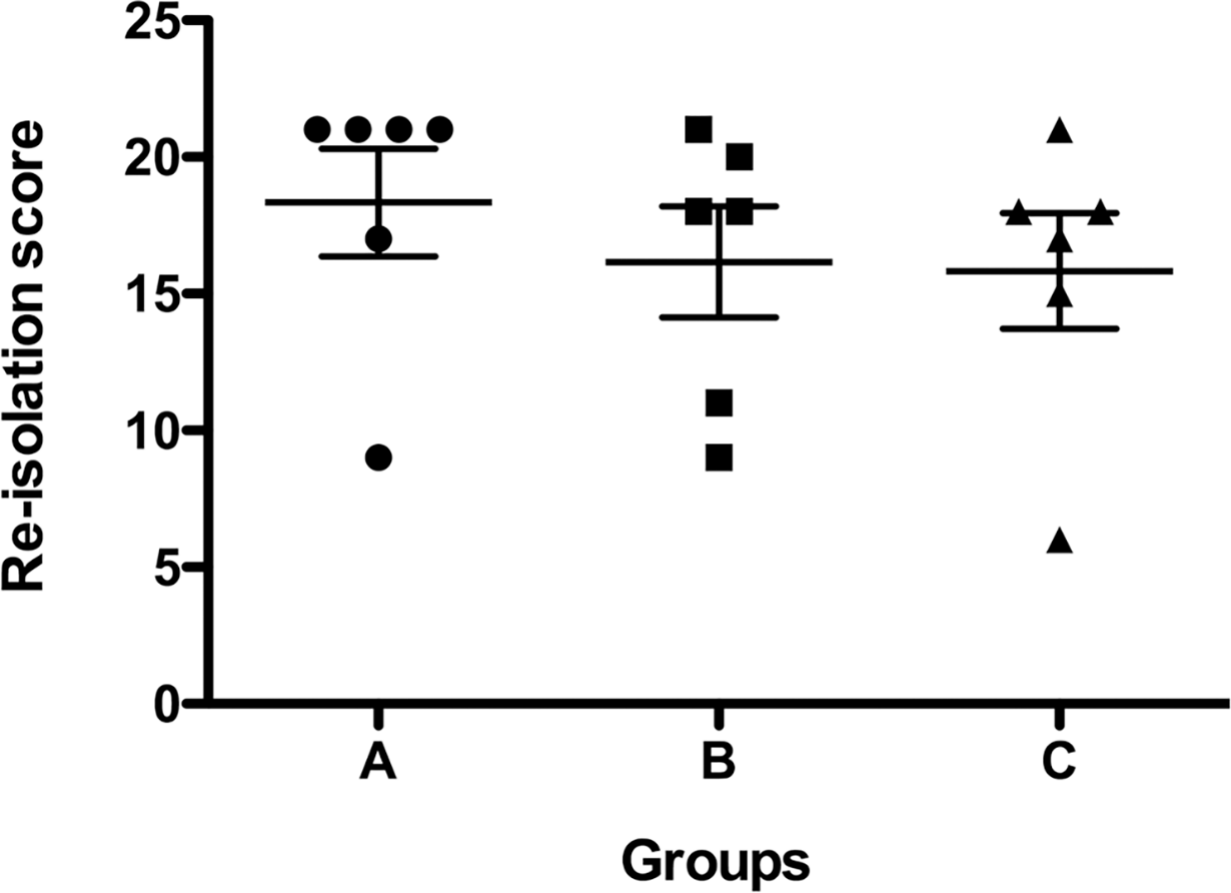
Semi-quantitative re-isolation of *P*. *aeruginosa* from the pig lungs at necropsy.

Re-isolation of *P*. *aeruginosa* was assessed from all 7 lobes of each pig at necropsy. *P*. *aeruginosa* was isolated from almost all segments of the lung in different densities. The *P*. *aeruginosa* contamination of the right lung was clearly less than on the left side, but similar in all groups. Accordingly, the *P*. *aeruginosa* re-isolation score in the LLL represented the highest score obtained in all animals. There were no significant differences in re-isolation score between the groups (Fig 5).

### Histopathology

**Fig 6:**
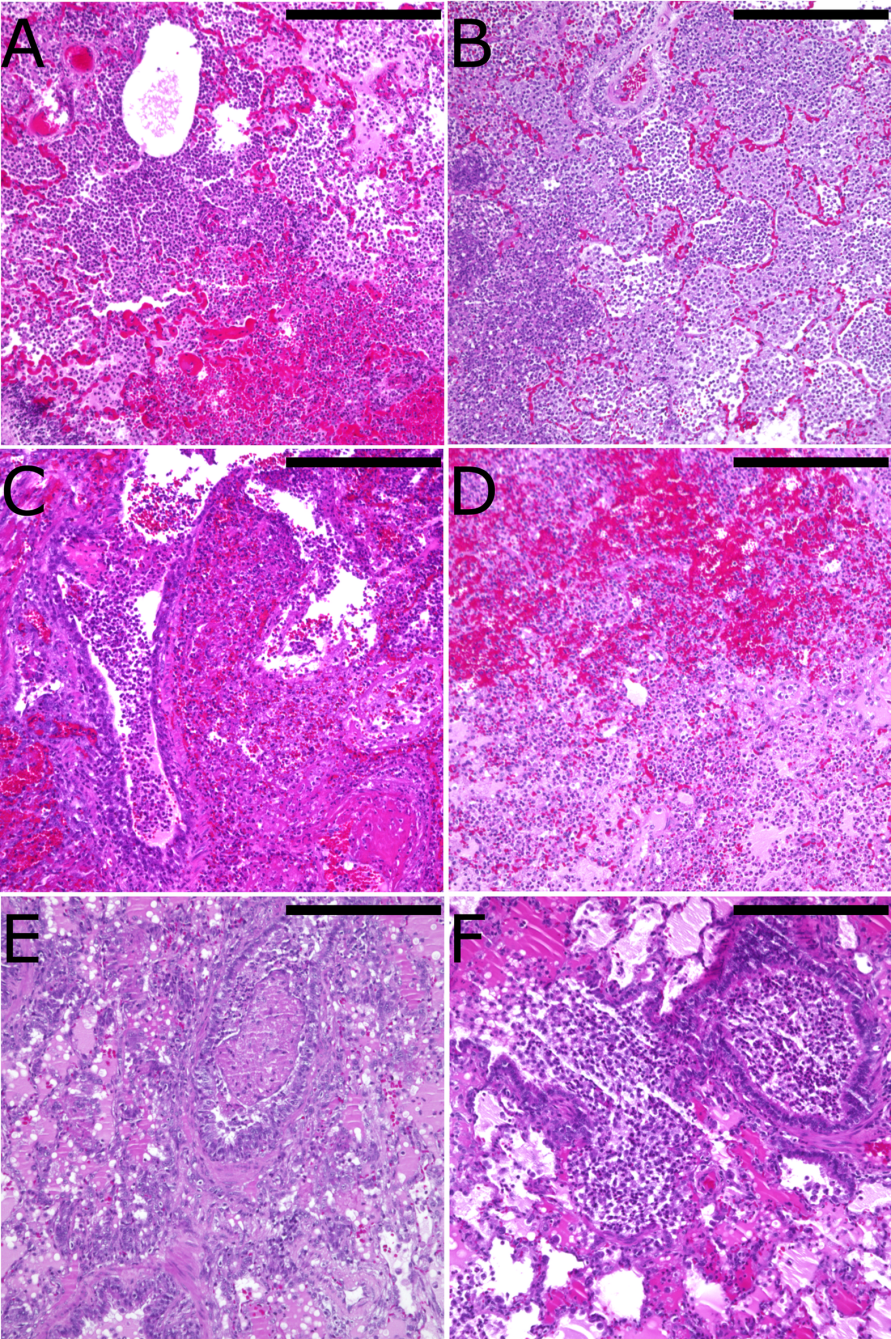
Histopathology of the *P*. *aeruginosa*-infected lung 4 days post-treatment. H&E staining of Group A (no treatment; panels A and B), Group B (i.v. antibiotic; panels C and D), and Group C (EVLP with high dose antibiotic; panels E and F). Scale bar = 250 μm.

Five days after infection or at the time of animal sacrifice, bronchopneumonic infiltrates could be observed in all animals. Histologic evaluation of the inflammatory response in the lungs did not reveal significant differences between Group C and Group A or B (Fig 6).

## Discussion

This experimental study was designed to explore the potential of ex vivo treatment for lung infections involving multidrug resistant bacteria. Using a large animal model of pneumonia, we have demonstrated that ex vivo high dose antimicrobial therapy followed by autotransplantation can be used to treat bacterial lung infections. EVLP allows us to specifically treat lungs with high doses of antibiotics without collateral drug toxicity to other organs. A recent study by Nakajima et al. [29], also performed EVLP with high dose antibiotics and found that this effectively reduced bacterial numbers. Our pilot study significantly extends these findings, showing that ex vivo treated lungs could be re-transplanted and resulted a higher rate of survival and a reduction in clinical symptoms as compared to conventional i.v. antibiotic therapy.

Several milestones were reached. In our preliminary experiments, we optimized the infection model described by Ferarri and Lu by adding cortisone for immunosuppression and infecting with a higher dose of *P*. *aeruginosa* to trigger highly reproducible, acute, severe pneumonia in miniature pigs[22,30,31]. We established a setup that enabled the treatment of single lungs in the EVLP system, although the system was originally designed for bilateral lung usage in the context of donor organ transplantation. During preservation of donor lungs with EVLP, there is usually enough tissue from the donor graft to connect the organ to the EVLP system. We implemented our recently described method of single-side autologous transplantation to mimic the potential future clinical application and performed the entire procedure within the same animal. We also customized the pre-, peri- and post-procedural management of the pigs to enable a complex surgical procedure lasting 10 to 12 hours without the option of intensive care support and prolonged mechanical ventilation. Despite their severe pneumonia and the prolonged surgical procedure, the pigs recovered well post-operatively with only very limited options for therapeutic and diagnostic interventions.

The evaluation of the pathomorphological alterations in the lung tissue quantified by the lesion score showed an advantage of colistin therapy over no therapy, but no advantage of ex vivo therapy over i.v. therapy. A limitation of this scoring system is that it does not differentiate with regard to specific pathological changes related to sequelae arising from bacterial colonization. The score only provides a quantitative assessment of the extent of pathologic irregularities. In Group A and B, we found changes based on pneumonia-like consolidation, atelectasis, and edema limited to the focal infection area. The EVLP group also showed consolidation especially in the main focal area of infection in the apex of the LLL. We also found edema in the lungs of all animals of this group, and in two cases, an embolism was found in the left superior lung vein. The edema is probably a result of difficulties in adequate fluid management for the pigs during the 10 h operation and the follow-up period.

The re-isolation scores showed no significant reduction of *P*. *aeruginosa* in the lungs in any of the groups. In the EVLP group, *P*. *aeruginosa* infection was detectable in the left lobe, which had been treated ex vivo. This raises the question of why the pigs in Group C showed a better outcome than those in the control groups. One factor might be the semi-quantitative re-isolation score we used[28], in which the maximum value is given when the second streaking of the primary sample is positive for *P*. *aeruginosa* and no further quantitative differentiation is undertaken. Another contributing factor might be that the animals in Group B received daily i.v colistin while the EVLP group did not receive systemic colistin treatment before or after the operation and therefore only received only a single dose of colistin. It is therefore possible that while bacterial colonization was reduced upon EVLP, *P*. *aeruginosa* in the left lung lobe had increased again over the four days post-procedure. In our setup, we used 100x the standard i.v. dosage (200 μg/ml), which translates to five times the 100% lethal in vitro dosage[10]. In preliminary in vitro experiments, it was determined that a two hour incubation time of *P*. *aeruginosa* with 200 μg/ml colistin was more than sufficient for 100% killing (data not shown). However, the parenchymal penetration, and consequently, the tissue concentration of colistin during the current EVLP therapy is not known. In addition, the extent of tissue infection remaining after a two hour perfusion of colistin is also not known. Tissue samples were not taken from the lungs during the EVLP run to avoid parenchymal or vascular defects with the corresponding risk of pneumothorax and bleeding upon re-implantation of the treated lung. To study these aspects in detail, future experiments will be performed, but instead of re-implantation, tissue samples will be collected and colistin concentration and bacterial colonization will be determined. For clinical applications, evaluation following EVLP to confirm clearance of the infection will be necessary before autotransplantation is performed. One thing that is not clear is whether the interruption of the lung’s systemic blood supply via the bronchial arteries after autotransplantation might be detrimental for post-operative antibiotic clearance of the infection. This possibility has to be investigated in further experiments focusing on the long-term outcome. In addition, it would be interesting to compare EVLP to in situ, normothermic, isolated single-lung perfusion (ILuP). Since ILuP is a less invasive alternative, it will be necessary to prove potential advantages of EVLP in further experiments. One likely major advantage of EVLP over ILuP is the washout of endotoxins that are released due to high-dose antibiotic therapy. Building upon this pilot study, in the future we hope to improve the ex vivo therapy regime to obtain better clearance of infection using longer ex vivo perfusion times and different combinations of antibiotics. For example, it was recently shown that colistin resistance could be overcome when used in combination with other antibiotics[32]. And lastly, future experiments could address whether nebulized colistin in combination with high dosage i.v. colistin treatment is beneficial as this seems to be a promising approach to improve bacterial eradication in the bronchial system and therefore may improve the clinical outcome[33].

In conclusion, in this proof-of-concept pilot study, we were able to demonstrate the possible advantage of high dose colistin therapy in comparison with traditional i.v. therapy with a clinical dose of colistin. Furthermore, this study also demonstrates the possibility of successfully performing autologous lung transplantation in animals with severe pneumonia. We proved the feasibility of ex vivo antimicrobial treatment of infection, in cases of otherwise incurable, life-threatening multidrug resistant infections. Moreover, these experiments lay the ground for possible future use of EVLP systems for different ex vivo therapeutic approaches.
